# Adherence to Guidelines among Italian Urologists on Imaging Preoperative Staging of Low-Risk Prostate Cancer: Results from the MIRROR (Multicenter Italian Report on Radical Prostatectomy Outcomes and Research) Study

**DOI:** 10.1155/2012/651061

**Published:** 2012-05-15

**Authors:** Alchiede Simonato, Virginia Varca, Mauro Gacci, Paolo Gontero, Ottavio De Cobelli, Massimo Maffezzini, Roberto Salvioni, Marco Carini, Andrea Decensi, Vincenzo Mirone, Giorgio Carmignani

**Affiliations:** ^1^“Luciano Giuliani” Department of Urology, University of Genoa, 16132 Genoa, Italy; ^2^Department of Urology, “L. Sacco” Hospital, 20175 Milan, Italy; ^3^Department of Urology, University of Florence, Careggi Hospital, 50134 Florence, Italy; ^4^Department of Urology, University of Turin, 10125 Turin, Italy; ^5^Departement of Urology, European Institution of Oncology, 20141 Milan, Italy; ^6^Department of Urology, E.O. Ospedali Galliera, 16128 Genoa, Italy; ^7^Department of Genitourinary Oncology, Urologic Oncology, Fondazione IRCCS Istituto Nazionale dei Tumori of Milan, 20133 Milan, Italy; ^8^Medical Oncology Unit, E.O. Ospedali Galliera, 16128 Genova, Italy; ^9^Department of Urology, University Federico II of Naples, 80131 Naples, Italy

## Abstract

*Objective*. A number of evidence-based guidelines for diagnosis and management of prostate cancer have been published. The aim of this study is to evaluate the adherence of Italian urologists to the guidelines concerning the preoperative imaging staging of prostate cancer. *Methods*. In October 2007 a multicentric observational perspective study called Multicentric Italian Report on Radical prostatectomy Outcome and Research (MIRROR) was started in 135 Italian urology centers. Recruitment was closed in December 2008 and 2,408 cases were collected. In this paper we have taken into consideration all examinations carried out for preoperative imaging staging, evaluating compliance with the recommendations in the American Urological Association (AUA) and European Association of Urology (EAU) guidelines. *Results*. Five hundred sixty-seven (53.34%) patients were not managed according to the EAU guidelines concerning T-staging, 545 (51.27%) concerning N-staging and 757 (71.21%) concerning M-staging. According to AUA guidelines, we also analyzed patients with a Gleason grade of biopsy specimens of 7: 238 (57.35%) of these patients had undergone testing for T staging, 244 (57.35%) for N-staging and 322 (77.60%) for M-staging. *Conclusions*. The compliance of Italian urologists with the guidelines is low, leading to an inappropriate increase in cost of care and unnecessary anxiety for the patients.

## 1. Introduction

Radical prostatectomy (RP) is an operation which is routinely performed in all Italian urology centers; the indications for this operation vary, and the preoperative workup may also vary from center to center and according to the stage of the disease.

The American Urological Association (AUA) and the European Association of Urology (EAU) have set up, and frequently review, the guidelines to provide urologists with an evidence-based pathway to diagnosis and management of prostate cancer. Concerning the AUA guidelines [1], there are no specific indications concerning imaging for patients undergoing radical prostatectomy.

The PSA best practice statement of the AUA guidelines [2] reports that routine imaging staging and bone scans are not necessary in every case of diagnosed prostate cancer. In particular, imaging is thought unnecessary if the PSA is <25 ng/mL and bone scans are not recommended when PSA is <20 ng/mL.

The EAU guidelines on prostate cancer were first published in 2001 [3] and since then have undergone several updates, up to the latest version in 2011 that can also be downloaded online [4]. According to EAU guidelines, [4] local staging (T-staging) should be based on findings from digital rectal examination (DRE) and in very specific cases from magnetic resonance imaging (MRI), even if the literature shows a wide range in the accuracy of T-staging by MRI, from 50–92% [4]. The assessment of lymph-node status (N-staging) can be avoided in patients with stage T2 or less, PSA < 20 ng/mL and a Gleason score ≤6, as they have less than a 10% likelihood of having node metastases.

A bone scan to rule out skeletal metastases may not be appropriate in asymptomatic patients if the serum PSA level is less than 20 ng/mL in the presence of well or moderately differentiated tumors. The purpose of the guidelines is not to prescribe how a clinician should treat a patient, but rather to provide a guide and an authoritative reference on the most appropriate diagnostic pathway currently available. It is expected that the majority of urologists will incorporate the guideline recommendations into clinical practice. To our knowledge, there are few articles that discuss the proportion of patients receiving appropriate use of imaging for CaP, none of them consider Italian population [5–9]. In the present work, we attempted to assess the adherence to these guidelines in several Italian urology centers.

## 2. Material and Methods

An independent multicenter perspective observational study called MIRROR (Multicenter Italian Report on Radical prostatectomy Outcome and Research) was begun in Italy in October 2007 with the aim of creating a register of the radical prostatectomies carried out, independently of the surgical technique used, collecting as many data as possible on a digital electronic Case Report Form (e-CRF) held by an independent third party Clinical Research Organization (CRO) Clicon srl of Reggio Emilia, Italy. The study was promoted by Leading Urological No profit foundation Advanced research (LUNA), the research foundation of the Italian Urological Association (SIU) which supported the study with an unrestricted grant.

The study involved 135 Italian centers which enrolled 2,408 consecutive patients who were chosen for and then submitted to radical prostatectomy. All the data were blind-recorded and stored in a web data base.

To ensure the standardization of this data collection, each center was provided with the same e-CRF. All data were stored in eXtensible Markup Language (XML) data files that were sent to the data server, recorded in a MicroSoft Structured Query Language (MS-SQL) database, and analyzed by a centralized data manager. The recruiting centers were either high-volume institutes or small community hospitals with a low number of radical prostatectomies per year, which enrolled only few patients. The average number of patients treated in each center was 35 with a range from 2 to 186. In this paper, we have taken into account the tests carried out for staging during preoperative evaluation in the various centers and correlated them with the recommendations of the AUA and EAU guidelines. According to EAU guidelines, patients with PSA <20 and Gleason score <6 can avoid preoperative imaging staging. The AUA guidelines, on the other hand, say that imaging staging is unnecessary if the PSA is <20 ng/mL and Gleason score is <7.

## 3. Results

We identified 1,288 patients overall with PSA <20 and Gleason score <6. Among these, we were able to evaluate 1,063 patients. As far as the other 225 patients concern, we do not have data concerning on preoperative staging so we excluded them from the analysis. The average age was 63.2 ± 6.5 years (median 62), ranging from 43 to 74. Preoperative PSA was 6.5 ± 7.2 ng/mL, median 5,8 g/mL. All patients undergone DRE and as regards the clinical stages, 956 patients were classified as T1 (89.9%) and 107 as T2 (10.1%). Preoperative prostate needle biopsy was performed transperineally in 307 (28.9%) patients and transrectally in 756 (71.1%). The Gleason grade of biopsy specimens was 4 in 34 patients (3.19%), 5 in 121 (11.38%), and 6 in 908 (85.42%). A centralized pathological review was not performed. The patients' characteristics are summarized in Table 1.

567 patients (53.34%) were not managed according to the guidelines concerning T-staging, and 545 (51.27%) patients were not managed according to the guidelines concerning N-staging. The most frequently performed test was an abdominal CT. Finally, 757 (71.21%) patients underwent a bone scan and, therefore, M-staging which is not recommended by the guidelines for this subset of patients.

**Figure 1:**
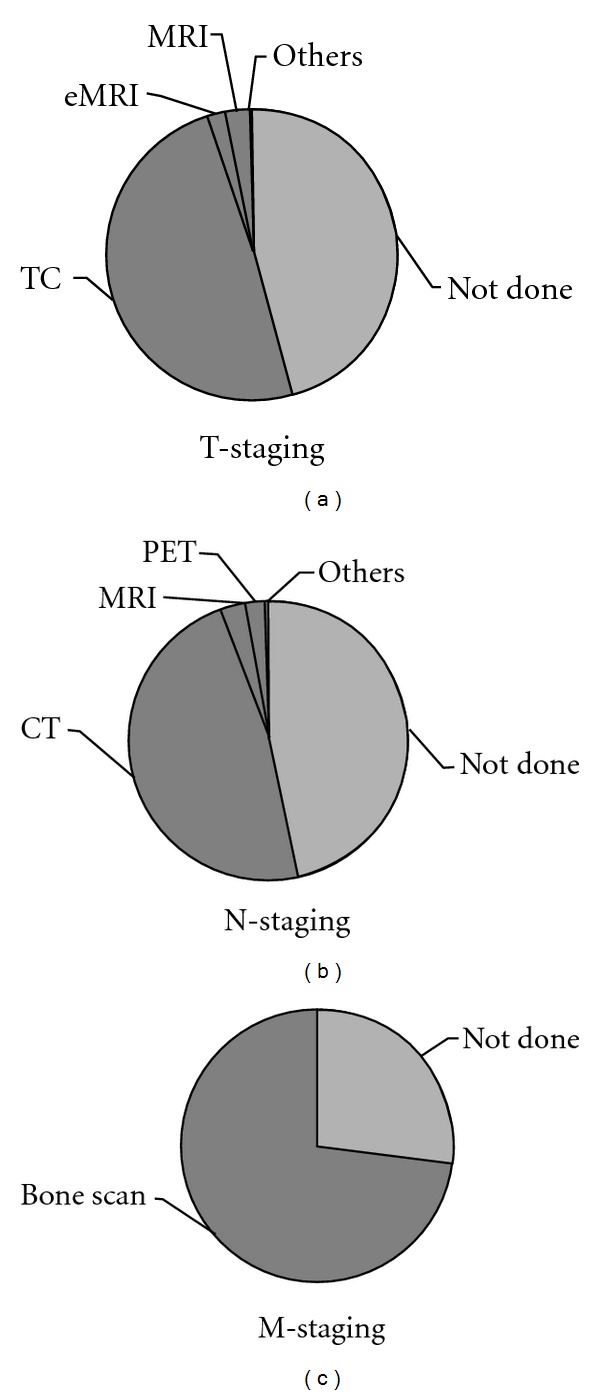
Proportion of patients with Gleason ≤7 and PSA ≤20 that underwent each examination.

As prescribed by AUA guidelines, we also analyzed patients with a Gleason grade of biopsy of 7. The average age of these patients was 65,4 ± 6,4 years (median 67), ranging from 41 to 77. Preoperative PSA was 7,7 ± 3,95 ng/mL, mediana 8,2 ng/mL. (Table 2). Among those 415 patients, 238 (57,35%) underwent testing for T staging and 244 (57,35%) for N-staging. Also in this subgroup of patients the most frequently performed test was an abdominal CT. 322 patients (77,60%) underwent a bone scan for M-staging. (Table 3). If we consider all patients with PSA ≤20 and Gleason ≤ to 7 overall, more than 50% of the patients underwent unnecessary testing for T- and N-staging and more than 70% for M-staging (Table 3, Figure 1).

As showed in Table 4, we did not report any significant difference in the use by Italian urologists of imaging procedures for TNM staging between EAU and AUA guidelines. Regarding the geographic distribution of these patients, we found that there was no difference in the percentage of patients undergoing unnecessary tests in high- or low-volume centers, including universities and nonuniversity centers, or among the various regions of Italy.

## 4. Discussion

The purpose of the guidelines in general and, therefore, also of the EAU and AUA guidelines is not to be prescriptive as to how a clinician should treat a patient, but rather to provide a guide and an authoritative reference on the most appropriate clinical pathway currently available.

Clinical practice guidelines are considered good tools for controlling and improving the effectiveness and efficiency of medical care [10]. However, it is a common concern that the publication of guidelines does not necessarily influence clinical practice. Passive dissemination is generally ineffective in changing physicians' behavior [11].

The data gathered in the course of our work are impressive: over 50% of patients underwent unnecessary T and N-staging and over 70% received unnecessary imaging for M-staging and we tried to analyze the reasons for this.

In literature, there are only few articles that examine the usage rate of various imaging modalities in patients diagnosed with prostate cancer, and to our knowledge all articles are related to American people [5–9].

In an analysis of the CaPSURE database, Cooperberg et al. reported that after 1997, 23% of low-risk prostate cancer patients received some form of imaging staging test [5].

In a more recent paper, Lavery et al. found that nearly half of low-risk patients underwent imaging that was not recommended by evidence-based guidelines [6].

A deeper analysis conducted by Choi et al. shows a widespread overuse of imaging for low-risk prostate and also a significant geographic variation in use. In particular, men living in areas of greater income were more likely to undergo imaging for low risk disease. They suggest this may be a result of increased patient demand, better access to imaging modalities, and more generous supplemental insurance [7].

Speculating on the reasons why the guidelines are not followed or only partially followed, even in important referral centers, one could argue that in Italy many urologists do not believe that guidelines, even if based on evidence and generated by an authoritative source, are actually able to reflect patients' individual situations. In literature, there are two references that go “head to head” in assessing the quality of the guidelines and their relevance for improving health [12–14]. Grol et al. argue that even when evidence is available, the final recommendation often reflects the personal opinions, local culture, or vested interests of the guideline developers [12]. On the other hand, Grol et al. affirm that guidelines are a repository of information for the clinician, recommending the use of treatments that have been proven effective and not using treatments that are ineffective and may be harmful [12]. In an article of Briganti et al. on the external validation of the currently available guidelines concerning the need to perform a bone scan in patients with newly diagnosed prostate cancer, it is concluded that guidelines are mainly based on limited, noncontemporary studies and that none of the studies and guidelines have yet been externally validated [15]. Defensive medicine is perhaps another reason why many urologists in Italy do not follow the guidelines for prostate cancer regarding preoperative tests. Defensive medicine is commonly defined as the ordering of treatments, tests, and procedures primarily to help protect the physician from liability rather than to substantially further the patient's diagnosis or treatment [16, 17]. While perhaps not “unnecessary” care, defensive medicine is meant to offer economic and psychological benefits to the physician rather than to the patient.

Defensive medicine may supplement care (e.g., additional testing or treatment), replace care (e.g., referral to another physician or health facility), or reduce care (e.g., refusal to treat particular patients). Some practices, herein termed *assurance behavior *(sometimes called “positive” defensive medicine), involve supplying additional services of marginal or no medical value with the aim of reducing adverse outcomes, deterring patients from filing malpractice claims, or persuading the legal system that the standard of care was met. Other practices, herein termed *avoidance behavior *(sometimes called “negative” defensive medicine), reflect physicians' efforts to distance themselves from sources of legal risk [18]. In our case, it is especially the first type of defensive medicine that comes to the fore, and it seems to us that this is not merely wasteful, but that such practices may also reduce access to care for other patients.

Another explanation for noncompliance with guidelines could also be that the guidelines are not popular and not largely adopted in Italy. Urologists probably know that such guidelines exist, but in everyday practice they are not consulted, probably due to limited time and pressure of work, so that guidelines do not enter into common clinical practice.

A further cause of noncompliance could be the inclusion of patients in clinical trials that prescribe other, additional testing. At the time of the enrollment, all the centers taking part in the study declared that they had no other ongoing trials.

Further, it may be that some doctors tend to prescribe a great number of tests even when these are not strictly necessary, in order to reassure the patient and to establish a doctor-patient relationship that in their opinion seems more solid.

Finally, another explanation could be a conflict of interest. In other countries, physicians may benefit financially from ordering more bone scans or CT scans, particularly if they have ownership interests in imaging centers or own their own CT scans. However, with the way the health system is structured in Italy, there should be no conflict of interest because most hospitals are public, and the physician prescribing or performing the tests does not receive compensation directly from the patient or according to the number of such tests.

Last but not least, the present study was not designed to calculate the additional costs that can result from unnecessary diagnostic workups, but it appears undeniable that prescribing unnecessary costly examinations such as a CT or a bone scan increases the financial burden on the national health system. Moreover, the problem of health costs in Italy is quite complex because each region has different pricing guidelines and the cost of each examination or test will usually vary from region to region. The value given is, therefore, an average based on the result of our inquiries. The complete abdominal CT with contrast medium costs 263,40 euros, the complete abdominal MRI with contrast medium costs 505,70 euros and the average cost of a total body PET is 1053,55 euros. Finally, a bone scan has an average cost of “only” 59 euros for each patient.

This is a prospective study which enrolled only patients undergoing radical prostatectomy and, therefore, we do not have information about the diagnostic pathway of patients undergoing other forms of therapy or active surveillance.

The lack of a central review of the slides could be seen as another limitation. For this reason, all the data entered in the e-CRF were assumed to be valid, including the biopsy Gleason score of 4-5 which is no longer considered valid in many countries.

## 5. Conclusions

The MIRROR study shows that the international guidelines are at present only partially observed by Italian urologists. The important effect of this is an unnecessary and inappropriate increase in cost of care, radiation exposure, and, possibly, unnecessary anxiety for the patients. The reasons for noncompliance may vary, but they should be a matter for study and reflection by the office boards of scientific urological associations.

## Figures and Tables

**Table 1:** Clinical data and preoperative characteristics of the 1,063 patients.

Clinical data	Age (mean ± SD/range)		63.2 ± 6.5 (43–74)
	BMI (mean ± SD/range)		26.5 ± 3.0 (19.2–33.0)
Preoperative characteristics	PSA (mean ± SD/range)		6.5 ± 7.2 (2.2–18.7)
	Clinical stage no. (%)	cT0-cT1c	956 (89.9%)
		cT2	107 (10.1%)
	Biopsy technique no. (%)	transperineal	307 (28.9%)
		transrectal	756 (71.1%)
		4	34 (3.19%)
	Gleason grade of biopsy no. (%)	5	121 (11.38%)
		6	908(85.42%)

**Table 2:** Clinical data and preoperative characteristics of the three groups of patients.

	Gleason ≤ 6	Gleason = 7	Gleason ≤ 7
AGE			
(mean ± SD/range; median)	63.2 ± 6.5 (43–74); 62	65.4 ± 6.4 (41–77); 67	64.3 ± 6.4 (41–77); 64
PSA			
(mean ± SD/range; median)	6.5 ± 7.2 (2.2–18.7); 5,8	7.7 ± 3.95 (0.13–19.66); 8,2	7.83 ± 3.74 (0.13–19.66); 9,8
CLINICAL STAGE			
cT1a-cT1c	956 (89.8%)	159 (38.3%)	1115 (75.4%)
cT2	107 (10.2%)	256 (61.7%)	363 (24,6%)
SURGERY			
RRP	865 (81.4%)	324 (77.97%)	1189 (80.4%)
ROB	136 (12.8%)	61 (14.77%)	197 (13.3%)
LAP	62 (5.8%)	30 (7.3%)	93 (6.3%)

**Table 3:** Preoperative imaging staging among patients who could avoid it.

	Gleason ≤ 6; PSA ≤ 20 (*n* = 1063)	Gleason 7; PSA ≤ 20 (415)	Gleason ≤ 7; PSA ≤ 20 (1478)
T-staging:	567 (53.34%)	238 (57.35%)	805 (54.76%)
CT	514 (90.8%)	213 (89.50%)	727 (90.31%)
eMRI	26 (4.5%)	8 (3.36%)	34 (4.22%)
MRI	26 (4.5%)	14 (5.88%)	40 (4.97%)
CT+MRI	1 (0.2%)	1 (0.42%)	2 (0.25%)
CT+eMRI	0	1 (0.42%)	1 (0.12%)
MRI+eMRI	0	1 (0.42%)	1 (0.12%)
N-staging:	545 (51.27%)	244 (58.80%)	789 (53.67%)
CT	499 (91.5%)	209 (85.66%)	708 (89.73%)
MRI	25 (4.6%)	14 (5.74%)	39 (4.94%)
PET	20 (3.7%)	14 (5.74%)	34 (4.31%)
CT+PET	1 (0.2%)	5 (2.04%)	6 (0.76%)
MRI+PET	0	1 (0.41%)	1 (0.13%)
CT+MRI	0	1 (0.41%)	1 (0.13%)
M-staging:	757 (71.21%)	322 (77.60%)	1079 (73.40%)
Bone scan	757 (100%)	100%	100%

**Table 4:** Difference in the use by Italian urologists of imaging procedures for TNM staging between EAU and AUA guidelines (*P* value calculated by chi-square Test).

	Gleason 7; PSA ≤ 20	Gleason ≤ 7; PSA ≤ 20	Chi Square Test
Number of Patients	**415**	**1470**	
T-staging	**238**	**805**	*P* value: N.S.
CT	213	727	
eMRI	8	34	
MRI	14	40	
CT+MRI	1	2	
CT+eMRI	1	1	
MRI+eMRI	1	1	
N-staging	**244**	**789**	*P* value: N.S.
CT	209	708	
MRI	14	39	
PET	14	34	
CT+PET	5	6	
MRI+PET	1	1	
CT+MRI	1	1	
M-staging	**322**	**1079**	*P* value: N.S.
BONE SCAN	322	1079	
